# Radiation therapy-induced aortoesophageal fistula: a case report and review of literature

**DOI:** 10.1093/gastro/gou081

**Published:** 2014-11-06

**Authors:** Malav P Parikh, Muhammed Sherid, Sreelakshmi Panginikkod, Harsh A Rawal, Venu Gopalakrishnan

**Affiliations:** 1 Department of Internal Medicine, Division of Gastroenterology and Hepatology, Presence Saint Francis Hospital, Evanston, Illinois, USA; 2 Department of Gastroenterology and Hepatology, Georgia Regents University, Augusta, Georgia, USA; 3 Department of Internal Medicine, Government Medical College Calicut, Kerala, India

**Keywords:** gastrointestinal hemorrhage, aortoesophageal fistula, radiation therapy, transthoracic endovascular aortic repair

## Abstract

Aortoesophageal fistula (AEF) is a rare cause of massive upper gastrointestinal hemorrhage. Thoracic aortic aneurysm, esophageal foreign body, esophageal cancer and post-surgical complications are common causes of AEF; however, AEF induced by radiation therapy is a rare phenomenon and seldom described in the literature. It is a catastrophic condition which requires rapid implementation of resuscitative measures, broad-spectrum antibiotics and surgical or endovascular intervention. Transthoracic endovascular aortic repair (TEVAR) is a newer and less invasive technique, which helps to achieve rapid hemostasis in patients with severe hemodynamic instability and offers advantages over conventional repair of the aorta in emergency situations. However initial TEVAR should be followed up with a more definitive surgical repair of the aorta and the esophagus, to lower the mortality rate and achieve better outcomes. We describe here a case of a seventy-year-old male who presented with massive upper gastrointestinal bleeding due to AEF induced by radiation therapy, and his subsequent successful initial management with TEVAR.

## Introduction

Aortoesophageal fistula (AEF) is a rare but life-threatening cause of massive upper gastrointestinal bleeding, which was first described by Chiari in 1914. Classical clinical features of AEF are described as ‘Chiari’s triad', which comprises mid-thoracic pain, initial sentinel arterial hemorrhage and final exsanguination hours to days after an asymptomatic interval [1].

## Case presentation

A 70-year-old male presented to the emergency department with mild chest discomfort and a brief episode of syncope. He was sweating profusely and appeared pale. Upon examination the patient was hypotensive, with blood pressure of 70/40 mmHg and tachycardic with a heart rate of 140 beats per minute. Soon after initial evaluation, he vomited approximately 400–500 mL of bright red blood and subsequently had four more episodes of massive hematemesis. The patient was intubated for airway protection and resuscitated with intravenous fluid, packed red blood cell transfusions and platelet transfusions.

Five months prior to the above presentation, the patient was diagnosed with carcinoma of the lung, with metastasis to the left hilar lymph nodes, for which he underwent chemotherapy and radiation therapy specifically directed towards left hilar lymph nodes. Shortly after completing radiation therapy, he developed gastroesophageal reflux and severe dysphagia, for which a percutaneous endoscopic gastrostomy (PEG) tube was put in place. A follow-up computerized tomography (CT) scan of the chest, obtained a day before the current admission, showed disruption of the distal esophageal wall and inflammation of the surrounding aortic wall.

**Figure 1.**
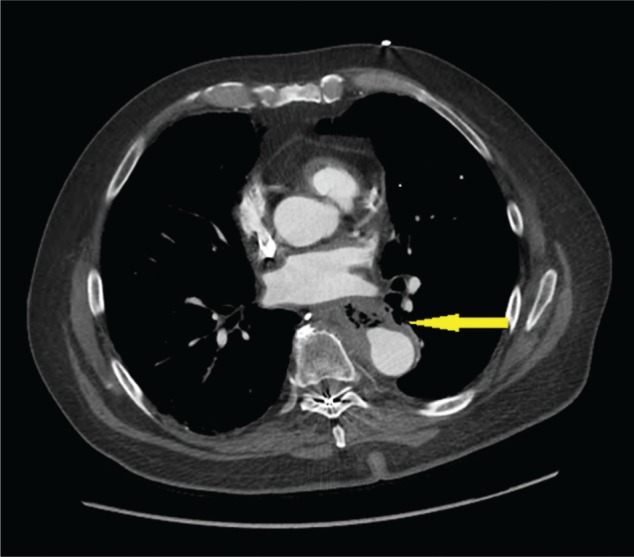
CT angiography of the chest. The arrow shows out-pouching of the intravenous contrast within the descending thoracic aorta, with marked mural thickening of the adjacent esophagus, with extra luminal foci of air between the esophagus and the aorta.

**Figure 2.**
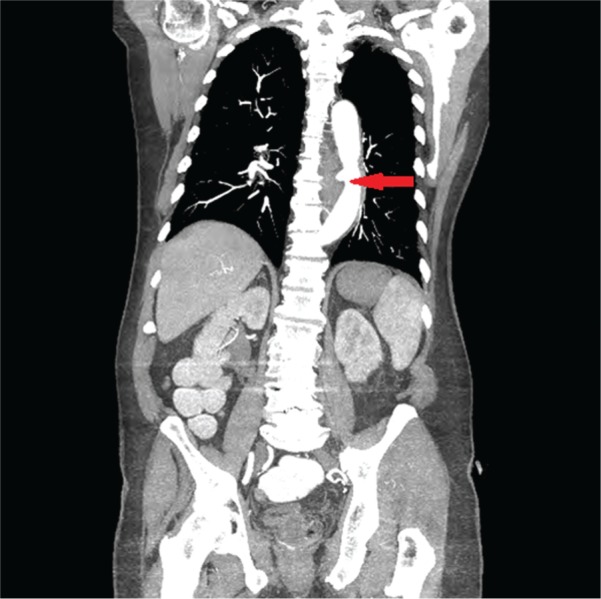
CT angiography of the chest and abdomen (coronal section). The arrow shows out-pouching of the intravenous contrast within the descending thoracic aorta, with marked mural thickening of the adjacent esophagus, with extra luminal foci of air between the esophagus and the aorta.

**Figure 3.**
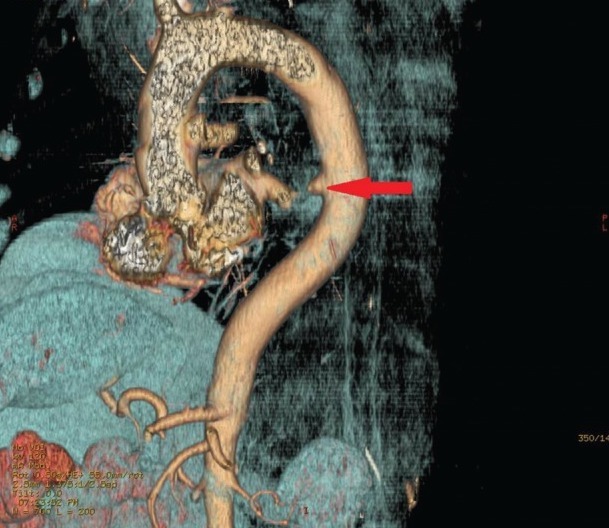
3D reconstruction image. The red arrow shows out-pouching of the descending thoracic aorta.

**Figure 4.**
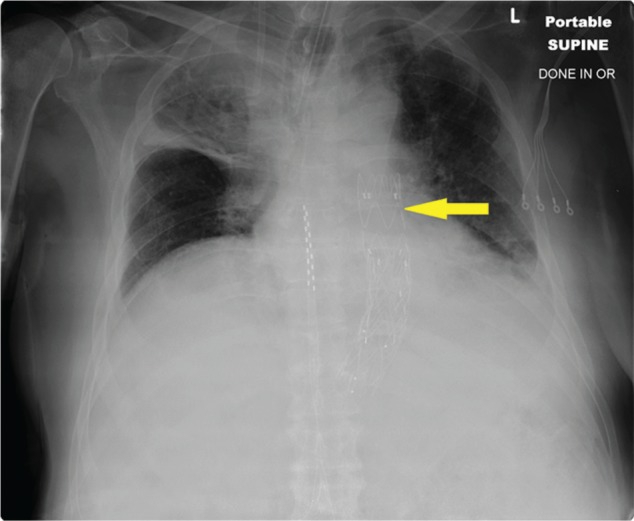
Follow-up chest X-ray after repair of aorta with transthoracic endovascular stent graft.

Based on this information, AEF was suspected and a CT angiogram of the chest and abdomen was obtained, which showed out-pouching of the intravenous contrast within the descending thoracic aorta, directed antero-medially towards the esophagus, and marked mural thickening of the adjacent esophagus with extra luminal foci of air between the esophagus and the aorta (Figures 1–3). Given the life-threatening nature of the disease and the patient's worsening clinical status, AEF was repaired with a thoracic endovascular stent graft system (Figure 4) and the patient was started on broad-spectrum antibiotics. He recovered uneventfully and esophageal stent placement was planned for a later date.

## Discussion

Thoracic aortic aneurysm, ingestion of a foreign body, esophageal malignancy and post-surgical complications are the most common causes of AEF. According to a review of 500 cases of AEF, esophageal malignancy was the third most common cause, showing the association between AEF and malignant intrathoracic neoplasms [2]. Review of the literature also showed a small number of cases in which radiation therapy was thought to be the primary causative agent for development of AEF [3–5]. Radiation-induced damage to the vessel wall is well recognized and reports suggests that AEF may be caused by radiation therapy alone or concurrent chemoradiation therapy (CCRT) [6]. Sivaraman *et al*. described a case similar to ours, in which a patient receiving radiation therapy for lung cancer developed an AEF with no evidence of residual tumor on autopsy, proving a definitive role of radiation therapy in the development of AEF [4]. Gabrail *et al*. reported a patient with esophageal cancer, who developed AEF after pre-operative CCRT, where chemotherapy was thought to act as a radio sensitizer, which potentiated the effect of radiation on the aortic wall [7]. Um *et al*. described a patient who developed AEF after receiving chemo-irradiation for non-small cell lung cancer and subsequent esophageal stent placement [6].

In general, side-effects of radiation therapy depend upon the field of irradiation, radiation dose in each session, total radiation dose and general medical condition of the patient [8]. Large arteries are not significantly affected by radiation, whereas small arteries and arterioles show sloughing of the endothelial cells, intimal fibrosis, thrombosis and subsequent occlusion. The *vasa vasorum* is a network of such small blood vessels supplying nutrients to the walls of major blood vessels. These small blood vessels can become occluded or thrombosed due to radiation therapy, resulting in perforation of the major blood vessels [3, 5]. In our case, the patient was diagnosed with metastatic lung cancer; however, on imaging, there was no evidence of direct tumor invasion of the esophagus or the aortic wall. Also the patient's radiation therapy was primarily directed towards the left hilar group of lymph nodes, which is anatomically in close proximity to the esophagus and aorta. Hence we believe that, in our case, AEF resulted primarily due to radiation therapy.

Management of AEF is determined, based on the hemodynamic stability and general clinical condition of the patient. Thoracic endovascular aortic repair (TEVAR) is the preferred approach in the initial management of patients with severe hemorrhagic shock [9–12]; it was first introduced in 1996 for the treatment of a fistula between the aorta and an adjacent organ [13]. It is a faster and a less invasive technique, allowing rapid control of blood loss in patients with hemodynamic instability [14]. It is also recommended in patients with relatively stable hemodynamics, as it allows for esophageal reconstruction and debridement of the contaminated field before attempting the open aortic repair, lowering the risk of graft infection [9].

It is important realize, however, that TEVAR does not definitely repair the AEF and that it should only be used as a ‘bridge' until a definitive surgical intervention is performed. Recent studies have shown an increased risk of mediastinitis, recurrent fatal bleeding and a high rate of mortality if TEVAR is used as the sole modality of treatment [10–12]. Hence all patients who have undergone TEVAR should be closely followed-up and considered for a definitive surgical correction of the esophagus and the aorta when they are deemed to be in appropriate health for surgery. A recent study involving forty-seven patients with AEF showed that esophageal repair—with esophagectomy and open surgical repair of the aorta (with prosthesis or homograft) and greater omentum wrapping—significantly improved the mid-term mortality rate of patients with AEF [10]. Antibiotics also play an important role in controlling infection and prolonged antibiotic treatment has a strong negative association with mortality [12]. According to a recent study by Mosquera *et al*., involving patients with AEF and aortobronchial fistula, hemodynamic instability, sepsis and conservative management were the most important risk factors for in-hospital mortality. The underlying etiology of the AEF predicted the long-term survival and infectious and malignant etiologies were associated with worst outcomes [15].

In summary, for a patient presenting with AEF, initial management with TEVAR, followed by open surgical repair of the aorta and the esophagus, along with appropriate antibiotic use, are associated with lower mid-term mortality and better outcomes.

*Conflict of interest statement*: none declared.
