# Radiation therapy-induced aortoesophageal fistula: a case report and review of literature

**DOI:** 10.1093/gastro/goy035

**Published:** 2018-09-11

**Authors:** Malav P Parikh, Muhammed Sherid, Sreelakshmi Panginikkod, Harsh A Rawal, Venu Gopalakrishnan

**Affiliations:** 1 Department of Internal Medicine, Division of Gastroenterology and Hepatology, Presence Saint Francis Hospital, Evanston, Illinois, USA; 2 Department of Gastroenterology and Hepatology, Georgia Regents University, Augusta, Georgia, USA; 3 Department of Internal Medicine, Government Medical College Calicut, Kerala, India

*Gastroenterology Report*, 2016, 4(2): 165–167. https://doi.org/10.1093/gastro/gou081.

The author of the above paper wishes to notify the reader that in the original article Figure 3 appeared incorrectly. The Figure appears correctly below. None of the findings or conclusions were affected by this error. The paper has been corrected online.

**Figure 3.**
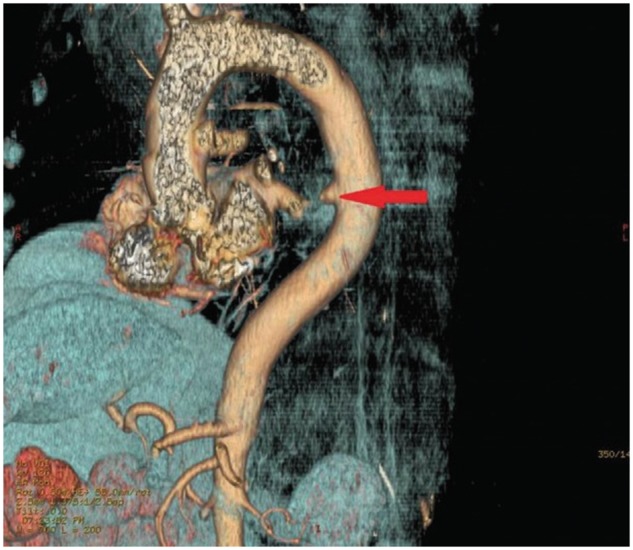
3D reconstruction image. The red arrow shows out-pouching of the descending thoracic aorta.

